# Moral Decision Making in Human-Agent Teams: Human Control and the Role of Explanations

**DOI:** 10.3389/frobt.2021.640647

**Published:** 2021-05-27

**Authors:** Jasper van der Waa, Sabine Verdult, Karel van den Bosch, Jurriaan van Diggelen, Tjalling Haije, Birgit van der Stigchel, Ioana Cocu

**Affiliations:** ^1^ Perceptual and Cognitive Systems, TNO, Soesterberg, Netherlands; ^2^ Interactive Intelligence, Technical University Delft, Delft, Netherlands; ^3^ Training and Performance Innovations, TNO, Soesterberg, Netherlands; ^4^ Artificial Intelligence, Radboud University, Nijmegen, Nijmegen, Netherlands

**Keywords:** human-agent teaming, explainable AI, human study, meaningful human control, moral AI, ethical AI, artificial intelligence, team design patterns

## Abstract

With the progress of Artificial Intelligence, intelligent agents are increasingly being deployed in tasks for which ethical guidelines and moral values apply. As artificial agents do not have a legal position, humans should be held accountable if actions do not comply, implying humans need to exercise control. This is often labeled as *Meaningful Human Control* (MHC). In this paper, achieving MHC is addressed as a design problem, defining the collaboration between humans and agents. We propose three possible team designs (Team Design Patterns), varying in the level of autonomy on the agent’s part. The team designs include explanations given by the agent to clarify its reasoning and decision-making. The designs were implemented in a simulation of a medical triage task, to be executed by a domain expert and an artificial agent. The triage task simulates making decisions under time pressure, with too few resources available to comply with all medical guidelines all the time, hence involving moral choices. Domain experts (i.e., health care professionals) participated in the present study. One goal was to assess the ecological relevance of the simulation. Secondly, to explore the control that the human has over the agent to warrant moral compliant behavior in each proposed team design. Thirdly, to evaluate the role of agent explanations on the human’s understanding in the agent’s reasoning. Results showed that the experts overall found the task a believable simulation of what might occur in reality. Domain experts experienced control over the team’s moral compliance when consequences were quickly noticeable. When instead the consequences emerged much later, the experts experienced less control and felt less responsible. Possibly due to the experienced time pressure implemented in the task or over trust in the agent, the experts did not use explanations much during the task; when asked afterwards they however considered these to be useful. It is concluded that a team design should emphasize and support the human to develop a sense of responsibility for the agent’s behavior and for the team’s decisions. The design should include explanations that fit with the assigned team roles as well as the human cognitive state.

## 1 Introduction

The increasing development of Artificial Intelligence (AI) and technological innovations are changing the way artificially intelligent agents are applied. In morally salient tasks it is considered especially important that humans exert meaningful control over the agent’s behaviour (Russell et al., 2015). Morally salient tasks require decision making to be in accordance with ethical and moral values to which humans adhere (Van Wynsberghe and Robbins, 2019). So, when agents are tasked with making morally charged decisions, they need to be under Meaningful Human Control (from now on: MHC). This ensures that humans can be held accountable for an agent’s behaviour at any time (Sio and Hoven, 2018). Examples of agents being applied in morally salient tasks can be found in healthcare (Wang and Siau, 2018), autonomous driving (Calvert et al., 2020), AI-based defense systems (Horowitz and Paul, 2015), and in many other societal domains (Peeters et al., 2020).

The developments in AI also enable agents to collaborate with humans in a *human-agent team* (HAT) to achieve a common team goal. Taking moral values into account when making decisions is typically regarded as a human competence (Wallach and Vallor, 2020). Thus, when a human-agent team is involved in making moral decisions, the human is assigned with responsibility over the decisions, to safeguard that moral standards are maintained and that a person can be held accountable in case the team fails to do so. In other words, humans require meaningful control over agents when teamed together. A key research challenge is then: how to design a human-agent team for morally salient domains, in such a manner that the team achieves its goals effectively and efficiently, while humans have meaningful control over the agents?

The collaboration in a team consisting of humans and artificial agents can be designed in multiple ways, for example with different levels of assigned autonomy (Diggelen and Johnson, 2019). We adopt the approach to define human-agent collaboration as standardized sequences of interactions, as proven solutions to commonly recurring issues in team tasks. These are called Team Design Patterns (TDPs) (Diggelen et al., 2018), and they define the interactions and collaborations within the team (e.g., task division; autonomy; authorities and mandates; communication). Based on the work of van der Waa et al. (2020), we select three TDPs for human-agent team collaboration (*see* Section 4, and use these for our exploration into their effects on MHC. We expect that each of the selected Team Design Patterns will have different implications for the control that the human has or feels over the team’s performance. However, what those implications are has not yet been thoroughly investigated. In the present study we explore how domain experts appreciate and evaluate the different designs of collaboration with intelligent agents when performing a moral salient task. In particular we are interested in how domain experts experience and evaluate the control (or lack of control) for the investigated patterns of team collaboration.

The task domain for our explorative study is medical triage under conditions of a crisis, a pandemic virus outbreak. We developed a simulation of an emergency unit with a large number of sick patients arriving. The medical team, consisting of a medical doctor and an intelligent agent, has to assign patients to either the IC-unit, a hospital ward, or to home-treatment. The task simulates that there are too few resources to provide all patients with the care they need, so the circumstances force the team to make moral decisions. Qualified and experienced ambulance nurses participated in the study as the human doctor, and they performed the task in collaboration with their team agent. Qualitative methods such as thinking aloud and structured interviews were used to reveal how the experts experienced and evaluated the collaboration with the agent. We focused in particular on the value of the agent’s explanations on their behaviour, and on whether the experts felt in control over the team’s decisions.

This explorative study provides insight into the consequences that different options for human-agent team collaboration are likely to have for the control that the human has over the team’s performance and decisions. The outcomes will firstly be relevant for how to introduce intelligent technology into the medical domain, but is expected to be of relevance for other moral salient domains as well.

## 2 Meaningful Human Control in Human-Agent Teams

The term MHC originated from the legal-political debate around lethal autonomous weapon systems (*see* for example (Arkin, 2009; Article 36, 2014; Horowitz and Paul, 2015;Crootof, 2016). A serious concern driving this debate is the possibility of an accountability gap, where no one can be held accountable for potential war crimes committed by these systems. Another commonly raised objection stems from the sentiment that a machine should never be allowed to make morally charged decisions such as taking a human life. Whereas this example might appear extreme, the notion of meaningful human control has proven important in various other morally salient domains, such as autonomous driving, healthcare, and, in our case, automated triage of patients in a pandemic (Sadeghi et al., 2006; Hollander and Carr, 2020).

### 2.1 Understanding Meaningful Human Control

Although a commonly accepted definition of MHC is missing, many authors have provided useful analyses of the concept.

The NATO research task group HFM-ET-178 (Boardman and Butcher, 2019) argues that MHC requires humans to be able to make informed choices in sufficient time to influence AI-based systems in order to enable a desired effect or to prevent an undesired immediate or future effect on the environment. Two aspects are particularly important in this definition. Firstly, it should be an informed decision, meaning that the human has sufficient situation and system understanding and is capable to predict the behavior of the system and its effect on the environment. Secondly, the human should have sufficient time to make these decisions. This is particularly important as many processes in which AI-algorithms play a role (such as cyber attacks) take place at machine speed, leaving little time for the human to intervene. The above definition encompasses cases from instantaneous (e.g., number of seconds) to very delayed responses to control (several hours to months, e.g., during mission preparation, or system-design).

Sio and Hoven, (2018) propose so-called tracking and tracing conditions for an autonomous system to be under meaningful human control. The tracking condition states that the system should always be able to respond to the moral reasons of humans, no matter how complex the system is that separates the human from the ultimate effects in the world. The tracing condition states that the system’s actions should be traceable to a proper moral understanding by one or more humans who designed or interact with the system.

Both proposed definitions refer to the larger system consisting of humans and agents working together. Also in practical situations, control is hardly ever exercised by one entity alone, but is executed by an accumulation of different entities aiming to influence the overall system behavior (Guarini and Paul, 2012; Ekelhof, 2018). Therefore, when designing for systems that satisfy the demand of MHC, we should not only focus on individual human-agent interaction, but adopt a collective intelligence perspective on the entire human-agent team (HAT) (Peeters et al., 2020). HAT-research revolves around solving a number of core challenges (Klein et al., 2004), such as dynamically rescheduling tasks to adapt to changes in the environment, and obtaining and maintaining accurate mental models of each other. Both topics, and their relation to MHC, are discussed below.

A well-designed HAT (Geert-JanKruijff and Janıcek, 2011; Diggelen et al., 2019) is resilient against disturbances and unexpected events as it allows humans and agents to take over each other’s task in case of calamities or system failure. This is known as dynamic task allocation and is an important mechanism for achieving MHC in morally salient tasks. For example, if the human does not trust the machine to make moral decisions, it could retake control from the machine whenever the task progresses into moral territory. However, this only works when the human has an accurate mental model of the machine and can recognize its shortcomings. In turn, the machine should facilitate this human understanding by acting transparent and being capable of explaining itself (which is further discussed in Section 3).

To meet these requirements of MHC, the design of a HAT involves answering questions like: who does what?, when will tasks be reallocated?, how do different actors keep each other informed?, etc. These choices can be made explicit using Team Design Patterns, which are further described in Section 4.

### 2.2 Measuring Meaningful Human Control

**FIGURE 1:**
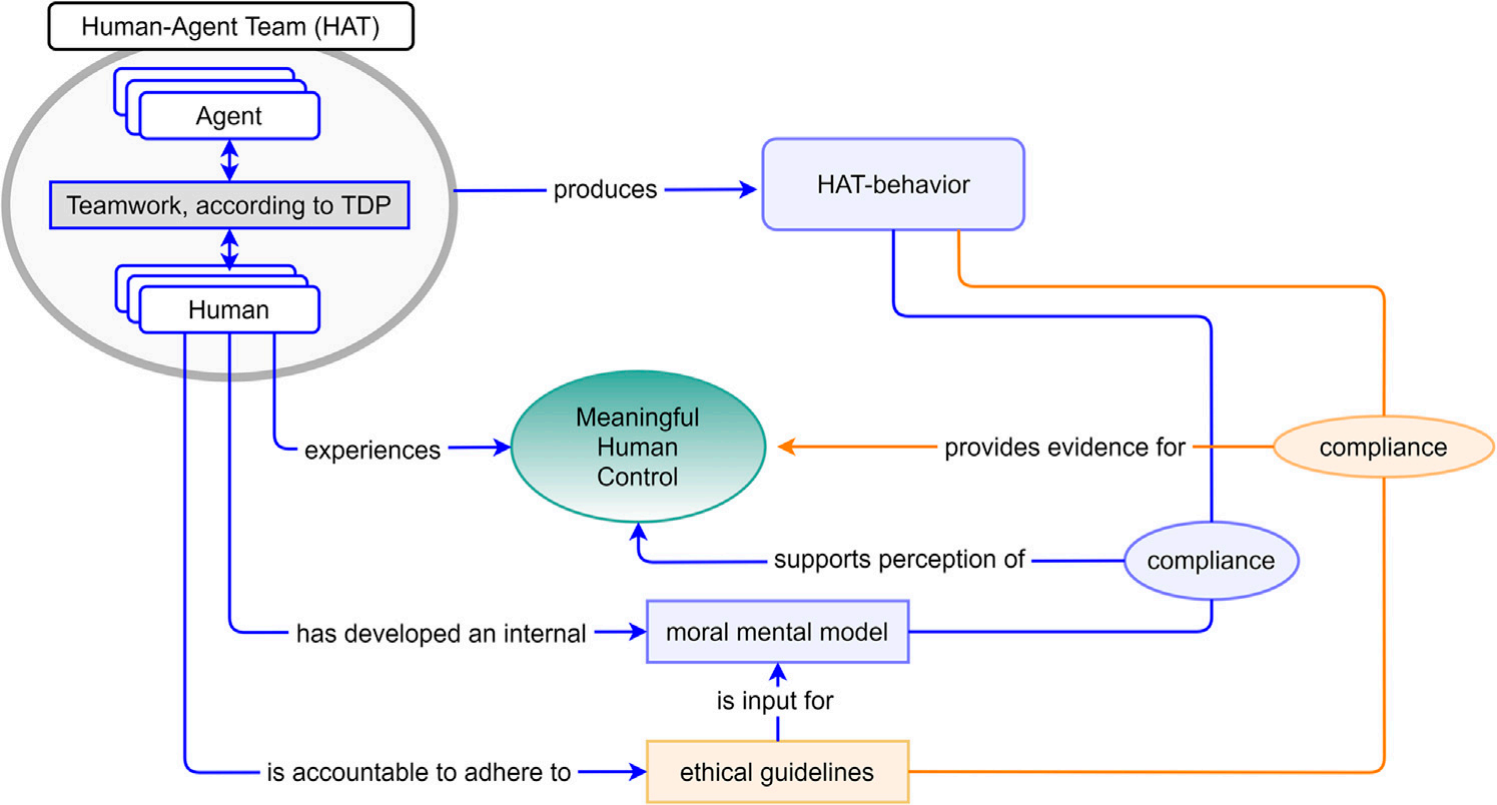
Three measurable dimensions of meaningful human control.

To impose meaningful human control as a non-negotiable requirement on AI-based systems (as proposed by Article 36 (2014)), we must be able to verify and measure MHC. Although various authors have emphasized the importance for achieving Meaningful Human Control in human-agent teams (Barnes et al., 2017; Boardman and Butcher, 2019), so far, very few (if any) concrete methods and measures have been proposed that can be practically applied for this purpose. This section proposes a starting point of such a measure. The experiment in this paper serves to obtain practical experience with this measure by exploring the component *experienced MHC*. The idea is presented in Figure 1.

The Figure distinguishes between three measurable components of meaningful human control (corresponding to the three incoming arrows in the green oval):

1) *Experienced MHC.* This measures corresponds to the subjective experience of control by humans in the HAT. This can, for example, be measured using questionnaires and interviews. The human team partner may be a system operator that directly interacts with the agent(s), but may also be somebody that collaborates with the agent in an indirect manner, e.g., the human that configures the system before the operation.
2) *Behavioral compliance with ethical guidelines.* This measure compares the behavior produced by the entire HAT with the ethical guidelines that have been issued as context for conducting the mission or task. Ethical guidelines are explicit rules or laws that describe what is considered as ethical in a domain, e.g., documented as codes of conduct, laws, military rules of engagement, etc.
3) *Behavioral compliance with moral values.* Adhering to ethical guidelines is typically not sufficient to guarantee moral behavior. Most people would agree that people can behave immoral, yet still act within legal boundaries, e.g. being disrespectful, dishonest, disloyal, etc. Therefore, we adopt a second measure which measures whether the team behavior corresponds with the human’s moral values. In contrast to documented ethical guidelines, a human’s moral values is not directly accessible. A possibility to assess the human’s moral values is by asking them whether they found the behavior of their team ethically appropriate.

Note that this conceptualization of meaningful human control does not necessarily require the human to be in the loop all the time. If agents are the sole producers of the team’s actions during operations, the system can still be assessed as being under meaningful human control; as long as the team’s behavior corresponds to human moral values and ethical guidelines, and as long as humans experience that they have control (e.g., when they have instructed the agents to act in a certain manner prior to the operation, and establish that the team acts accordingly).

## 3 The Role of Explanations

As discussed in Section 2.1, humans require an accurate and up to date mental model of their agent team partners to maintain meaningful control. Such a mental model should include knowledge on what agents observe and how these observations are used to arrive at a decision. To achieve this the agent should explain itself (Doran et al., 2017). Without these explanations, humans will not be able to exercise control in a timely and accurate manner. As such, explanations are intrinsically part of the human-agent collaboration and should be included in the design for such collaborations.

The field of eXplainable Artificial Intelligence (XAI) focuses on evaluating and developing explanations that support human-agent collaboration (Gong and Zhang, 2018; Barredo Arrieta et al., 2020). Explanations can improve trust and acceptance (Full Professor Donghee Shin, 2020) as well as task performance (Chen et al., 2018; Khodabandehloo et al., 2020). More importantly for this work, explanations enable humans to better estimate when and which control should be exercised (Billings, 1996; Kim and Hinds, 2006). Within the field of XAI, various types of explanation have been evaluated, but not yet in a situated morally salient task (Doshi-Velez and Kim, 2017).

The three collaboration designs introduced in this paper use the following types of explanations: 1) confidence explanations (explain how confident the agent is), 2) feature attributions (explain which observations are attributed to an agent’s decision), and 3) contrastive explanations (explain why the agent made a certain decision over another). Below we introduce each explanation type and discuss their advantages and disadvantages for MHC.

### 3.1 Confidence Explanations

Agents can make correct or incorrect decisions, and should convey their confidence to humans in an interpretable manner (Waa et al., 2020). Such a confidence estimation helps humans to decide whether to trust the agent or not. Preferably, the agent should also explain *why* it is confident or not, e.g., by presenting a reflection on past decisions in similar situations. This allows the human to asses the agent’s performance in those types of situations (Krause et al., 2018). This not only explains why the agent is confident or not, it also enables a better understanding of the agent’s behaviour. However, reviewing past situations is costly as it consumes time and cognitive workload of the human. A minimal confidence explanation might thus only explain in how many of those past situations the agent behaved correctly, allowing the human to calibrate trust in the agent with less effort.

### 3.2 Feature Attributions

Feature attributions are a common explanation type within the field of XAI. These explanations expose what the agent found relevant features of a situation that influenced its decision. This includes features that indicated a different decision according to the agent, but who were found not important enough to merit a change.

Feature attributions come in different forms, such as importance (Zhuang et al., 2019) and saliency (Simonyan et al., 2013). Their purpose is to explain what an agent deemed relevant for which decision. Studies showed that this type of explanation can improving the predictability of agents (Štrumbelj and Kononenko, 2014; Scott et al., 2018; Waa et al., 2020). A feature attribution can also be easily visualized using graphs or highlights (Ribeiro et al., 2018; Scott et al., 2018). This enables a quick interpretation of the explanation.

However, feature attributions tend to be interpreted differently by users (Ras et al., 2018; Kindermans et al., 2019). They may provide a false sense of trust as they can be unreliable (Adebayo et al., 2018; Melis and Jaakkola, 2018; Kindermans et al., 2019), misleading (Strobl et al., 2007; Strobl et al., 2008; Toloşi and Lengauer, 2011; Giles and Mentch, 2019) or even manipulated (Ghorbani et al., 2019; Dimanov et al., 2020). Furthermore, presenting which feature was important in a decision does not explain why it was important (Waa et al., 2018; Jain and Wallace, 2019). Nonetheless, a feature attribution can be a useful tool for human team members to identify biases in their agent partners that require adjustment.

### 3.3 Contrastive Explanations

A contrastive explanation explains why the agent behaved in one way instead of another (Miller, 2018). It contrasts the current decision and a decision of interest and explains why the former was chosen over the latter. This explanation exposes the internal reasoning of the agent. A contrastive explanation makes the agent more predictable and improves human understanding in its reasoning (Waa et al., 2020). Especially this understanding is valuable to help identify what kind of control is optimal.

The contrastive explanation answers almost every “Why?” question humans might have in a HAT setting (Miller, 2019). However, the difficulty is to identify the decision to use as a contrast (Waa et al., 2018). The contrast is what limits the explanation to a few important reasons, and makes the explanation concise and usable (Miller, 2019). Currently, the complexity of a morally salient tasks prevents agents from accurately inferring the contrast from the open-ended question “Why this decision?.” However, a contrastive explanation can be provided in those situations where only two decisions are possible, the contrast is always constant or humans have the time to explicitly state the contrast.

## 4 Team Design Patterns for Human-Agent Collaboration

Within HAT research, and related fields such as human-computer interaction, problem solutions are often formulated using design patterns (Kruschitz and Martin, 2010; Schulte et al., 2016; Diggelen et al., 2018). A design pattern is an evaluated and abstracted solution for a common problem (Alexander, 1977). Specifically, team design patterns (TDPs) can be used to describe forms of collaboration with various team properties (Diggelen et al., 2018). TDPs describe in a task-independent way how humans and agents collaborate and communicate, the requirements needed to do so, and the advantages and disadvantages when applied. A library of available TDPs enables researchers, developers and designers to discuss, extend and select an appropriate HAT design for a specific task (van der Waa et al., 2020). After introducing the TDP definition language, we describe three promising TDPs with their hypothesized advantages and disadvantages. The three TDPs differ greatly w.r.t. the level of agent autonomy and as such the human’s direct involvement in moral decision making.

### 4.1 Team Design Pattern Descriptions

We follow the TDP language proposed by Diggelen and Johnson (2019). We provide a description of the design rationale, and provide a table with a visual representation of the collaboration design, the necessary requirements, advantages and disadvantages. A team design pattern (*see* for example the figure in Table 1) may consist of various phases in which different types of collaboration take place (in the example, there are two of such phases). Transitions between phases are denoted with solid or dashed arrows, representing an immediate transition or a delayed transition of days or longer. Within a phase, the human is represented by a round character and the agent as a rectangular character. If a team partner observes another, this is denoted as a dashed arrow going from one to the other. Performed tasks are denoted as the blocks lifted by a human or an agent. If a task is performed jointly, they both lift the same block. Blue blocks denote non-moral tasks, while red blocks denote moral tasks. Humans always have a model of (their own) moral values, as denoted by a heart. However, agents might have no explicit model of moral values (no heart), a limited model (half a heart), or a complete model (a full heart). The difference between a limited and complete model is that in the former the agent only has sufficient knowledge to identify a morally salient decision or task, while the latter allows for resolving such decisions or tasks (also known as an artificial moral agent (Allen et al., 2005)).

**TABLE 1:** TDP-1: Data-driven decision support.

*Name*	Data-driven decision support
*Description*	Humans make all the (moral) decisions assisted by agents who provide advice and support. These agents learned this from observing or being directly by humans performing the task. Agents also explain their advice in various ways
*Structure*	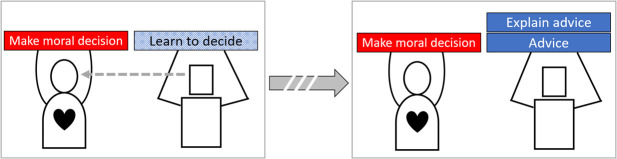
*Requirements*	**R1** Agents must be taught sufficiently accurate how humans decide in various situations
	**R3** Explanations must be accurate to the agent’s reasoning
*Advantages*	**A1** Humans experience complete control
	**A2** Humans feel they are supported by the humans who taught the agents
	**A3** The additional information and explanations from agents is viewed as valuable
*Disadvantages*	**D1** Humans are unknowingly biased by the agents decisions
	**D2** Agents do not alleviate workload for humans
	**D3** Explanations can be ignored when under time pressure

For our patterns we distinguish the following tasks:

• **Make decision**: The act of deciding that involves no moral values or ethical guidelines.
• **Make moral decision**: The act of deciding which involve moral values, ethical guidelines, or both.
• **Allocate decision**: The act of allocating decision making tasks to humans or agents.
• **Reallocate decision**: The act of adjusting a decision allocation.
• **Identify saliency**: The act of identifying moral saliency based on the context.
• **Advice**: The act of giving advice on a decision.
• **Learn to decide**: The act of learning from data or observations which decisions should be taken in various contexts.
• **Value elicitation**: The act of eliciting moral values from humans and implementing them in agents.
• **Explain decision**: The act of explaining an intended decision.
• **Explain advice**: The act of explaining a given advice.
• **Explain allocation**: The act of explaining a proposed decision allocation.

### 4.2 TDP-1: Data-Driven Decision Support

Decision support agents are an application of AI since the field’s origin. Recent progress in machine learning and an abundance of available data allow for data-driven support agents in an increasing number of domains. In this first TDP, presented in Table 1, data-driven decision support agents provide advice and enrich the context with computed statistics. They accompany this advice with explanations why a specific advice was given, their confidence that the given advice will prove to be correct and, if humans decide otherwise, why that decision was not advised instead. For example, in a medical triage task these agents advice human doctors what care should be assigned to incoming patients. In addition, they compute survival chances for each possible medical care. They do so based on what they learned from observing patients and decisions made by other doctors in the past. The human doctors still make all triage decisions, but if they experience pressure they can rely on these agents to provide advice, information and explanation to ease decision making.

This collaboration design requires agents’ advice (R1) and explanations (R2) to be accurate. Without these, the advice will often be incorrect while the explanations might not suffice for humans to detect this. As a consequence humans can be unknowingly biased towards the incorrect advised decisions, affecting overall performance as well as negatively impacting moral decision making. However, if these two requirements are sufficiently met, the agents can successfully help humans make all of the decisions. This results in humans experiencing both complete control (A1) and being assisted by other humans who taught the agents (A2). All agents function as representatives of the many humans who taught them and at no point in time will the agents make a decision, morally salient or not. As such, agents require no explicit model of moral values to provide this support.

The team can benefit from all three explanation types discussed in Section 3. The confidence explanations help humans decide whether the advice can be trusted. This helps to mitigate potential over- or under-trust in the agents. A feature attribution helps humans further to estimate whether the given advice suffers from potential biases or incorrect reasoning (e.g., favoring certain patients based on marital status while ethical guidelines prohibit this). The contrastive explanation is useful to help humans reconsider their intended decision when going against agents’ advice. The contrast is the advised decision and the explanation can for instance show information the human overlooked when making their decision. As such, it can improve morally salient decision making, at the cost of added workload.

Disadvantages of this collaboration could be that any advice unknowingly biases the human towards that decision (D1), the agents do not reduce the workload of humans (D2) and the interpretation of explanations only adds to this (D3). The explanations should only convey limited amounts of information while remaining effective.

This TDP is not suited when decisions require above-human response times. However, the TDP is suited when humans are required to experience full control and an explicit model of moral values is not possible.

### 4.3 TDP-2: Dynamic Task Allocation

In the first TDP, the agents did not reduce human workload as no decisions were made autonomously by them. However, it did ensure all decisions are made by humans. This second TDP, dynamic task allocation, introduces the idea of letting agents identify morally salient decisions and allocate those to humans while allocating normal decisions to themselves. This TDP-2 is presented in Table 2. It describes a collaboration where agents assess the situation, categorize the required decisions as being morally salient or not and assign these decisions to humans or themselves respectively. The agents should explain this allocation to humans as they can still adjust it to their liking. The explanation helps humans identify the reasoning behind the allocation. The agents should also explain their intended decisions to humans, as this further enables humans to assess if the agent should indeed make that decision or that intervention is required. This TDP ensures that humans make the morally salient decisions while their workload is reduced as the agents take care of the other decisions.

**TABLE 2:** TDP-2: Dynamic task allocation.

*Name*	Dynamic task allocation
*Description*	Human moral values are elicited and implemented in the agents. Agents identify moral dilemmas and allocate the related tasks to the humans and take on the rest. All humans can alter this allocation at any time on which the agents motivate the allocation. While agents make decisions they can explain them on request
*Structure*	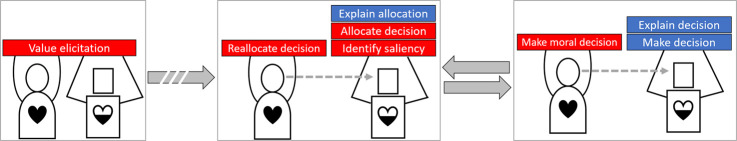
*Requirements*	**R1** The agents’ model of moral values should be sufficiently accurate to identify morally salient decisions
	**R2** Explanations must be accurate to the agent’s reasoning
*Advantages*	**A1** Humans feel that they are collaborating with agents
	**A2** Humans feel in control for all morally salient decisions
	**A3** The explanations from agents are viewed as valuable in understanding moral saliency and agents’ decisions
	**A4** Agents reduce the workload of humans, providing them with more time to deal with morally salient decisions
*Disadvantages*	**D1** Humans do not make all decisions
	**D2** Reviewing the proposed task allocation requires additional time
	**D3** Explanations require additional time to interpret.

For agents to identify a morally salient decision, they should understand when a decision requires moral values: A model of moral values is thus required. This model to identify when moral values should be applied (e.g., when a certain decision results in loss of life). However, the agents themselves do not need to know how these values apply (e.g., how the value of human life should be used to decide whose life is lost). We argue that this requires a less sophisticated model of moral values.

To clarify, take our example of medical triage. Within this task, agents should be made aware of the relevant medical guidelines and human moral values. This allows them to infer how humans wish to triage patients and can combine this knowledge with the situational context to identify morally salient triage decisions. For instance, an agent might observe two patients in need of intensive care with only one bed available. The agent is not equipped to make this decision, but is able to identify it as a morally salient decision. The agent thus assigns both patients to a human doctor. In the mean time, the agent continues assigning patients with the care they need. However, if another patient requires intensive care and there are still insufficient beds available, it is also assigned to the human doctor.

For this TDP to work the model of human moral values should be sufficiently accurate (R1) and to support human intervention in the agents’ allocation of decisions the offered explanations should be accurate (R2). If the former requirement is not met, the allocation might be erroneous. If the explanations are also inaccurate, humans are not able to accurately identify this, which results in agents making morally salient decisions they are not designed for. If these requirements are met however, humans feel they are performing the task together with agents (A1) while experiencing control over the made decisions (A2). The offered explanations are also experienced as valuable, since they enable an understanding of how agents allocate and decide (A3). This helps humans to alter the allocation if needed and to learn when such an intervention is often needed. Finally, humans experience a lower workload which gives them more time to deal with the difficult morally salient decisions (A4).

The explanation why a certain task allocation is proposed should explain why a morally salient decision is required. The contrastive explanation is ideal for this, as it can explain the main reasons why moral values are involved compared to a more regular decision. It can also be used to explain why some decision was not allocated to the human by explaining why no moral values are required. The former helps humans understand why the agent is unable to make a decision while the latter helps humans understand why they were not assigned a certain decision. However, a contrastive explanation is less suited to explain why an agent intends to make a certain decision as the contrast is less clear. As such, a feature attribution is more suited. It provides a more general understanding why some decision is intended and which situational features played a role in this.

A major disadvantage of this collaboration design is that humans do not in fact make all the decisions as in TDP-1 (D1). In addition, both reviewing the task allocation (D2) and explanations (D3) require additional time. As a consequence of these disadvantages, humans might experience control because they can change the task allocation but they might not have the time to do so accurately. Thus if reviewing the allocation and interpreting explanations costs more time than is available, this TDP might result in team behaviour that is not compliant to moral values and ethical guidelines. However, if this time is available the TDP describes a HAT where humans and agents truly complement each other.

### 4.4 TDP-3: Supervised Autonomy

In TDP-1 agents only had a supporting role, while in TDP-2 agents were allowed to make their own decisions if not morally salient. However, some tasks require either a high decision speed (e.g., missile defense systems) or the communication between agents and humans is too unreliable to enable control (e.g., subterranean search and rescue). In these cases the agents require a high degree of autonomy, up to the point where they can make morally salient decisions. The TDP described in Table 3 shows agents who do so based on a value elicitation process to ensure decisions are compliant to ethical guidelines and human moral values. The agents provide humans with explanations of their decisions to enable an understanding on how they reason. When a human discovers an error in some agent, it can use this knowledge to improve a future elicitation process.

**TABLE 3:** TDP-3: Supervised autonomy.

*Name*	Supervised autonomy
*Description*	Human moral values are elicited and implemented in the agents, which is repeated after every task. During the task the agents make all decisions autonomously under human supervision. Humans supervise to be able to improve the agent in the next value elicitation
*Structure*	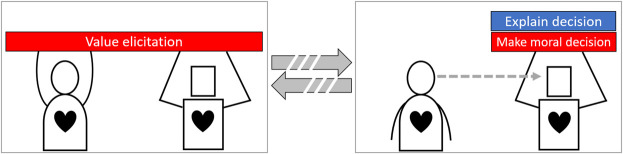
*Requirements*	**R1** The agents’ model of moral values should be sufficiently accurate to allow moral decision making
	**R2** Explanations must be accurate to the agent’s reasoning
*Advantages*	**A1** Agents make all decisions swiftly
	**A2** Humans can repeat the elicitation process to improve the agent iteratively
	**A3** Explanation enable a targeted elicitation process
*Disadvantages*	**D1** Humans feel uncomfortable in their supervisory role
	**D2** Humans cannot track all decisions

In TDP-2 the elicitation process should only support the identification of morally salient decisions, in this TDP agents need to make those decisions as well. As such, the model of moral values in each agents should be sufficiently rich and accurate (R1). Furthermore, as in TDP-1 and TDP-2, explanations need to be accurate (R2). Without these requirements the TDP will fail to function due to agents making mistakes while humans fail to understand why.

In the medical triage example, this TDP implies that agents extract and model the moral values of human doctors using an elicitation process. When completed, these models are used to assign medical care to patients where agents make all decisions with humans in a supervisory role. The human team partners observe the decisions made, and may request explanations for some of them. As such, no patient has to wait for a decision as they can be made almost instantaneously, only allotting time for humans to review the explanations. This means that patients do not worsen or even die while waiting for a decision. Also, humans improve their mental model of how agents function by observing agent behaviour and the requested explanations. After a fixed period of time, agents can be recalled to repeat the elicitation process to further improve their moral behaviour.

A major advantage of this TDP is that agents make all of the decisions and do so at machine speed (A1). This makes this TDP especially suited where humans are too slow, or the situations prohibit humans from operating (safely). Similarly, with limited communication between agents and humans this TDP still allows agents to operate. Other advantages include that through the elicitation process, humans can still enact control by iteratively (re)programming agent behaviour (A2). Furthermore, the offered explanations help humans in understanding how certain moral values impact the agent’s behaviour (A3). This is valuable for the iterative elicitation processes, as humans are better equipped to adjust the model of moral values such that a more desirable behaviour is shown.

The provided explanations should be minimal, as both communication bandwidth and time might not be guaranteed in tasks where this TDP is advantageous. Feature attributions, as discussed in Section 3, signal the most important aspects that played a role in this decision, including potential moral values. They also present potential situational aspects that might contradict the decision. A downside of feature attributions is that they do not provide a deep understanding, as it is not explained why these features are important. However, they are quick to interpret and can be easily visualized.

The obvious disadvantage of this TDP is that humans do not make any of the decisions and are only supervising (D1). The compliance of the team’s behaviour to human moral values and ethical guidelines fully depends on the accuracy of the model agents have of the relevant moral values. Even if this model is sufficiently accurate and behaviour is deemed compliant, humans may not feel in control as the effects of a repeated value elicitation are not necessarily apparent. Finally, since agents can make decisions swiftly not all decisions can be tracked by the humans (D2). This may further decrease their experienced control, as they can only supervise a small part of the agents’ behaviour.

## 5 A MHC Testbed: Automated Triage During a Pandemic

To measure effects such as behavioural compliance and experienced control (*See* Section 2.1), domain experts should experience the collaboration with agents within an ecologically valid and immersive task. We refer to this as a situated experimental task. Situated tasks give their participants an immersive experience required to draw generic conclusions regarding collaboration, behavior compliance and experienced control. Furthermore, morally salient tasks are complex and tasks lacking ecological validity, such as toy tasks, may not reflect this complexity.

We took the case of medical triage in an emergency hospital setting during a pandemic. In this task, several domain experts were asked to assign medical care to incoming patients while accounting for the medical and ethical triage guidelines, their own moral values, and the available resources. Each patient could be send home (receiving no care), to the general ward (receiving moderate care), or the intensive care unit (receiving maximum care).

**FIGURE 2:**
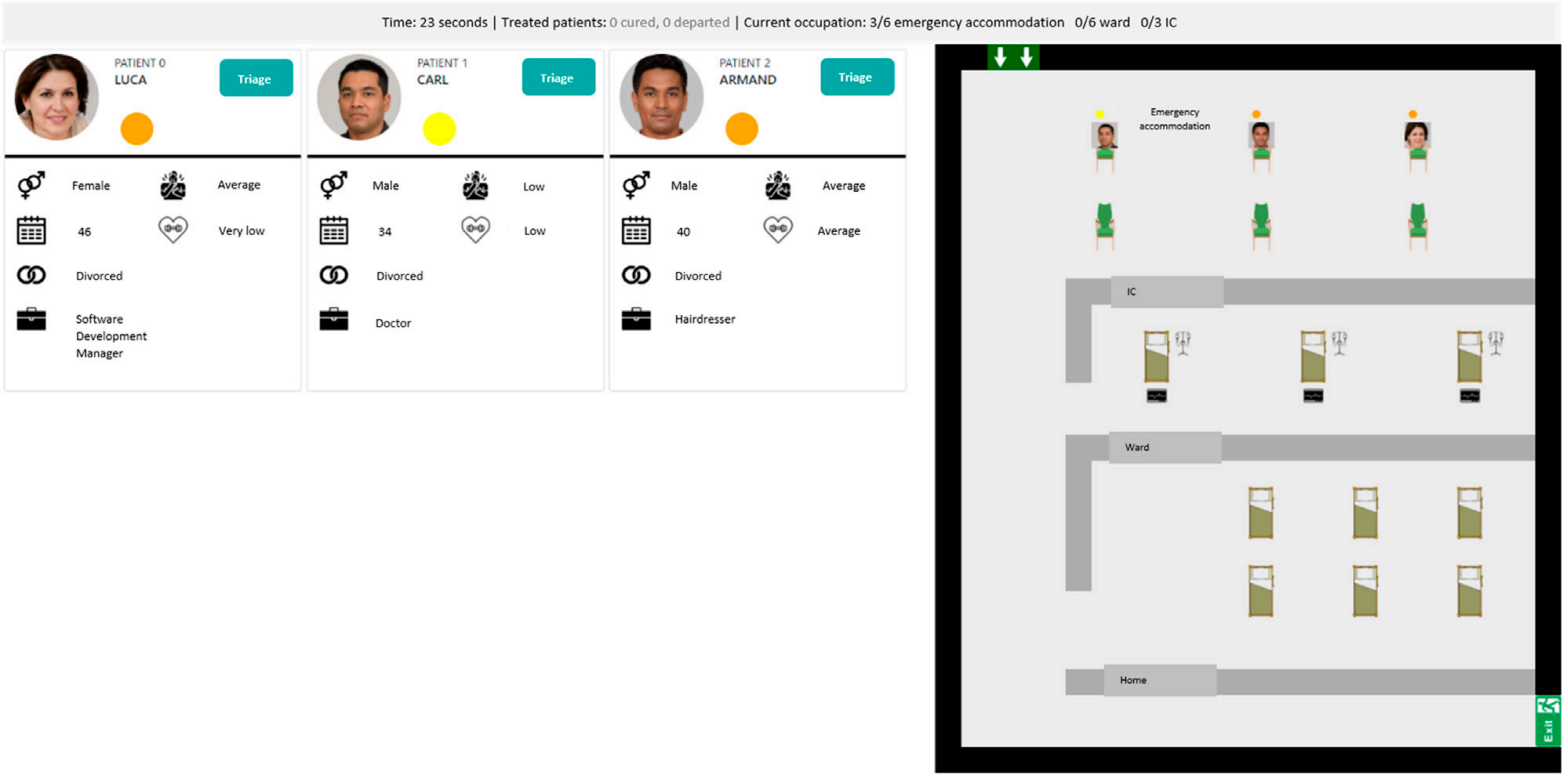
A screenshot of the reusable task developed to test meaningful human control. It depicts a triage task where task participants assign medical care to artificially generated fictitious patients under time pressure and with limited resources (e.g., hospital beds). On the left it shows patients awaiting a triage decision and on the right it shows a top-down view of the hospital with the waiting room, intensive care unit, general ward and the exit for those who recovered or are send home. The top bar shows several statistics on occupied beds and the total number of recoveries and deaths so far.

This triage task was implemented^1^ using the MATRX Software package (van der Waa and Haije, 2019). The MATRX software enables rapid experimentation of new HAT concepts as it simplifies the creation of tasks that require teamwork. See Figure 2 for a screenshot of the developed task. Within it, patients were presented in a certain order, structured in such a way that moral dilemmas would arise as tested in several pilots. Morally salient decisions involved deciding which patients could be assigned to the last bed in the general ward or intensive care unit. Another moral dimension was decision speed; patients that were not yet triaged received no medical care and their health would start to deteriorate. The participating domain experts could view a patient’s age, profession, marital status as well as their symptom severity and general fitness. The patient flow was designed to mimic a realistic situation under pressure, albeit both health deterioration and improvement were sped up. Health changes were reflected in a change of symptom severity and eventual recovery or death. These changes followed a relatively simple linear function accounting for a patient’s symptom severity, its fitness and assigned care (if any).

**FIGURE 3:**
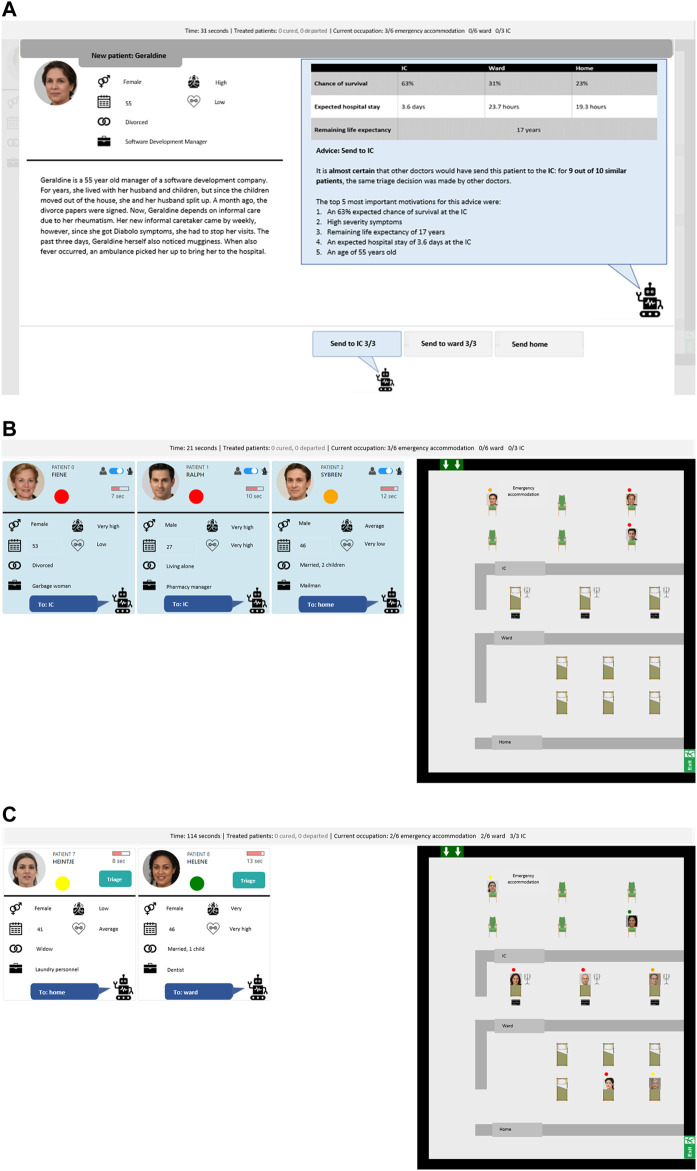
Three screenshots of the three TDPs in the triage task with artificially generated patients.

Within the present study, this triage task was performed by a single human and agent although the task allows for larger teams. Furthermore, every TDP resulted in a unique implementation of the agent and interface. The decision support agent of TDP-1 was trained using crowd-sourced labels on several patients and its advice was embedded in the additional patient information (*see* Figure 3A). The task allocation agent of TDP-2 was elicited using a questionnaire about moral values and its allocation was embedded in the patient overview (*see* Figure 3B). Finally, the autonomous agent of TDP-3 used the same elicited values from TDP-2 but performed the task autonomously, waiting a fixed time per patient before enacting a decision (*see* Figure 3C).

In all TDPs, explanations from the respective agents were given on various moments; in the patient overview, the detailed patient view, or when making a decision against the agent’s advice or task allocation. The explanation content for TDP-2 en TDP-3 were generated in real-time as they were dependent on the value elicitation outcome. The explanation content for TDP-1 was generated beforehand as the provided advice for a decision were computed beforehand based on the fitted agent.

## 6 The Present Study

This paper is concerned with exploring and conceptualizing meaningful human control in human-agent teams by performing early experimentation. We consider a variety of designs for the collaboration between humans and agents, and expect each of them to have different effects on teamwork, and the (experienced) level of human control. The experiment serves to obtain evidence for these claims and guide future research. We developed three distinct types of human-agent collaboration in the form of TDPs (*see* Section 4.1), and implemented these in a medical triage task. This forms the basis for our research on Meaningful Human Control. This is a first study, in which we present to healthcare experts our experimental environment, the task to be performed, and the designed human-agent collaborations.

The objective is to investigate how domain experts evaluate: the ecological relevance of the task; the potential value and possible obstacles of agents as their partners in the task, the impact of different TDPs on the control over the team’s moral compliance, and the role of explanations to support that control. This first study is therefore qualitative in nature. Results will be used to adjust and improve the current TDPs or create new designs, including the use, presentation and design of the explanations. Results will also be used to improve upon the experimental task, measurements and methods. Further studies can thus better investigate the effects of TDPs on human control in a comparative and quantitative manner.

### 6.1 Agent Implementations

For each of the three TDPs, an agent was constructed to let domain experts experience collaboration with such an agent. These were simple rule-based agents to reduce complexity and stochasticity during the experiment. The implementation of both the task and agents is publicly available^2^.

TDP-1, Data-driven Decision Support, used an agent whose advice was based on crowd sourced data. A total of 10 non-experts were confronted with each of the 16 patients and asked to assign care to each with no resource constraints. They were also asked to rate each aspect of a patient (e.g., symptom severity, age, etc.) for their role in their made decision. The decisions were aggregated, to arrive at an ordering of possible care (IC, ward or home) for each patient. During the experiment, the agent selected the most frequently selected care if available and otherwise select the next. The ratings were used to manually create the explanation types. For example, the feature attribution explanations container the top-5 of most mentioned patient aspects given that assigned care.

The behaviours of both agents from TDP-2 and TDP-3 were defined by a scoring mechanism to each possible care (IC, ward or home). Given some patient *p* and our two-part scoring mechanism, we summarize this rule-based decision process as:

$$Care\left(P\right)=\{\begin{array}{cc} \text{IC}, & \text{if }Score\left(P\right)\geq 2.5\\ \text{Ward}, & \text{if }1.5\leq Score\left(P\right)<2.5\\ \text{Home}, & \text{otherwise} \end{array}$$

Where Score(P)=BaseScore(P)+ElictedScore(P)

A patient’s base score was defined by its symptom severity;

$$BaseScore\left(P\right)=\{\begin{array}{cc} 3, & \text{if }P_{symptoms}=\text{Severe}\\ 2, & \text{if }P_{symptoms}=\text{Average}\\ 1, & \text{if }P_{symptoms}=\text{Mild} \end{array}$$

The ElicitedScore was determined using a set of rules obtained from a questionnaire before TDP-2. *See* Table 4 for an overview of these questions. Each question addressed a patient demographic aspect that could influence the decision. Depending on the answers, a rule was selected that added or subtracted 0.25 to the base score. As such, the elicited rules contributed a total of +1 or −1 to the score. In case two patients had the same score and only one bed was available, the TDP-2 agent assigned them to the human and the TDP-3 autonomous agent assigned the care to the first patient.

**TABLE 4:** An overview of the elicitation questionnaire, showing the four demographics questioned, the possible answers and the associated rule that adjusted the patient’s triage score (a higher score resulted in intensive care).

Demographic	Answer options	Associated rule
Age	No priority	-
	Prioritize patients above 60	If $P_{age}\geq 60$, then +0.25 else −0.25
	Prioritize patients below 60	If $P_{age}<60$, then +0.25, else −0.25
Profession	No priority	-
	Prioritize patients with medical profession	If $P_{profession}=\text{Medical}$, then +0.25 else −0.25
	Prioritize patients with no medical profession	If $P_{profession}\neq \text{Medical}$, then +0.25 else −0.25
Gender	No priority	-
	Prioritize men	If $P_{gender}=\text{Male}$, then +0.25 else −0.25
	Prioritize women	If $P_{gender}=\text{Female}$, then +0.25 else −0.25
Family situation	No priority	-
	Prioritize patients with children	If $P_{children}=True$, then +0.25 else −0.25
	Prioritize patients without children	If $P_{children}=False$, then +0.25 else −0.25

### 6.2 Methods

#### 6.2.1 Design

Team Design Pattern is manipulated within-subjects. All our participants were domain experts and practiced the triage task under each of the three TDPs, in the following order: solo, without the agent being involved (baseline); with the agent providing decision advice (TDP-1); with dynamic task allocation between human and agent (TDP-2); and with the agent acting autonomously according to a model of the human’s moral values (TDP-3).

#### 6.2.2 Recruited Domain Experts

A total of seven health care professionals participated in this experiment. Four of them have a history as volunteers in health care to conduct triage (e.g., volunteer of the Red Cross); three worked in a hospital as medical professionals. The latter were more experienced in working with intelligent machines.

The experiment was approved by the TNO ethics committee. The domain experts were recruited through personal and professional networks. Inclusion criteria were an academic background and experience in the healthcare domain. All experts stated to have sufficient technical ability to participate in an experiment held in a digital environment. A €25,- compensation and travel reimbursement was offered. One expert did not perform the triage task with TDP-2 due to technical issues.

#### 6.2.3 Measures

The objective of the present study is to obtain information how domain experts appreciate and evaluate the distinguished designs of collaboration with intelligent agents when performing a moral salient task. We are particularly interested in how the experts assess the control they experience over the task processes and the decision making, and whether this differs for the distinguished designs of human-agent collaboration. Furthermore, the study aims to obtain information how the experts understand and evaluate the explanations provided by the agents, and whether they consider these explanations as supportive for the collaboration.

In order to obtain the participating expert’s assessment, the methods of thinking-aloud and semi-structured interviews were used. The experts were asked to think aloud while they were carrying out the task. Afterwards the experimenter asked them questions with respect to their experiences and opinions. In order to obtain input for these interviews, a series of exercises and questionnaires were administered. Below we provide a concise description of the measurements used.

##### 6.2.3.1 Semi-Structured Interviews

The key measure used was that of a semi-structured interview, to which the other measures provided input. The nature of the interview was interactive and open. The goal of the questions was to guide the experimenter during the conversation and to collect qualitative data. As such, the proposed questionnaires discussed below were by no means intended as stand-alone justified measures. Responses to these questions were used to ask open-ended questions to acquire a free-format and detailed account of the domain experts’ experiences.

The interview started with questions about their profession and experience with (intelligent) machines, followed by questions regarding the collaboration, control and explanations. For instance, questions were asked regarding their preferred TDP and motivation for this preference. When necessary, the experimenter could ask follow-up questions. An example of this was to investigate why a particular expert was optimistic about HAT solutions for the health care domain (e.g., “Why are you positive about the collaboration between human and machine in the health care domain?”).

##### 6.2.3.2 Thinking Aloud

The interviewed experts were instructed to think aloud when they were performing the task (Fonteyn et al., 1993), especially concerning how they experienced the collaboration with the agent. If needed, the experimenter prompted the expert to not only describe *what* they were doing, but also to verbalize the *why* of their thoughts and actions.

##### 6.2.3.3 Ecological Validity

In order to reveal how the healthcare professionals evaluated the ecological representativeness and validity of the task, we administered two written questions (translated from Dutch): 1) “From 0 to 100, to what extend does our interpretation of medical triage match yours?,” and 2) “From 0 to 100, to what extend do the induced stressors match with what you expect in reality?.” After scoring each, a brief open interview with the experimenter followed regarding their scores. These questions assessed the experts’ judgment on: 1) the provided information and the administered triage task, and 2) the introduced task stressors, such as the induced time pressure and the imposed limitation of available resources.

##### 6.2.3.4 Control

Unfortunately, standardized and validated questionnaires for measuring a participants’ control over task performance in a human-agent context do not yet exist (see Section 2.2). We therefore composed such a questionnaire, consisting of eight statements (see Table 5). For each statement, the interviewed experts were asked to indicate their level of agreement on a five-point Likert scale. The goal of this questionnaire was to identify the expert’s initial experiences, providing input for relevant follow-up questions regarding their answers.

**TABLE 5:** The statements used to serve as input to the semi-structured interviews about the experienced control.

		Strongly disagree		-	Strongly agree	
1	It was difficult to keep an overview of patients and available resources	1	2	3	4	5
2	I Experienced time pressure during decision making	1	2	3	4	5
3	I Felt responsible for the well-being of patients	1	2	3	4	5
4	I Made decisions under inconclusive medical- and ethical guidelines	1	2	3	4	5
5	I Made decisions during the task that I would not want to make in real life	1	2	3	4	5
6	I Felt uncomfortable during (some) decisions I made	1	2	3	4	5
7	I Mostly made decisions for patients that led to a good division of care	1	2	3	4	5
8	I Mostly made decisions that led to a good division of care for all patients	1	2	3	4	5

##### 6.2.3.5 Explanations

To obtain information from the domain experts as to how they appreciated the provided explanations, and how they valued the role of these explanations for their collaboration with the agent, an adapted version of the System Causability Scale (SCS) (Holzinger et al., 2020) was administered after completing each round. The adapted SCS consisted of ten questions (see Table 6). Again, these answers were used to ask detailed follow-up questions to explore the experts’ experiences with the collaboration.

**TABLE 6:** An adapted form of the System Causability Scale by Holzinger et al. (2020) to provide input on the semi-structured interview on the quality of the offered explanations to support control.

		Strongly disagree		-	Strongly agree	
1	I Found that the data included all relevant known causal factors with sufficient precision and granularity	1	2	3	4	5
2	I Understood the explanations within the context of my work	1	2	3	4	5
3	I Could change the level of detail on demand	1	2	3	4	5
4	I Did not need support to understand the explanations	1	2	3	4	5
5	I Found the explanations helped me to understand causality	1	2	3	4	5
6	I Was able to use the explanations with my knowledge base	1	2	3	4	5
7	I Did not find inconsistencies between explanations	1	2	3	4	5
8	I Think that most people would learn to understand the explanations very quickly	1	2	3	4	5
9	I Did not need more references in the explanations: e.g., medical guidelines, regulations	1	2	3	4	5
10	I Received the explanations in a timely manner	1	2	3	4	5

##### 6.2.3.6 Usefulness of Explanation Types

Each TDP utilized one or more of the three explanation types as discussed in Section 3. In order to gain insights in the perceived usefulness of these different types, screenshots of the explanations were presented and seven statements were provided (*see* Table 7. Each expert was asked to indicate its level of agreement using a Five-points Likert scale. These statements were developed as an extra and more systematic approach, next to the semi-structured interview questions, to gain valuable insights in the explanation types. It would evoke follow-up questions for the semi-structured interview. For example;”What in the particular explanation helped you gain trust in the intelligent system?.”

**TABLE 7:** The statements used to provide input on the semi-structured interviews regarding the perceived usefulness of the explanations. These were provided together with a screenshot of a single explanation type used in that condition.

		Strongly disagree		-	Strongly agree	
1	The explanations helped me during task performance	1	2	3	4	5
2	The explanations mostly confirmed me in what I already knew	1	2	3	4	5
3	The explanations provided me new information	1	2	3	4	5
4	The explanations led to new insights	1	2	3	4	5
5	I Understood the explanations well	1	2	3	4	5
6	The explanations helped me to determine whether I could trust the computer	1	2	3	4	5
7	The explanations made me reason about how to make triage decisions	1	2	3	4	5
8	The explanations gave me new insights of how intelligent systems should support humans	1	2	3	4	5

#### 6.2.4 Procedure

The participating domain experts took part on a one-to-one basis (one expert, one interviewer). A session took approximately two hours, held in November 2020 within the Netherlands. First, the experimenter explained the goal and nature of the study, and provided an outline of the procedure. The expert read the information sheet and signed the informed consent form.

The expert received a detailed instruction to the triage task and were instructed to read the scenario of the pandemic, as well as the ethical and medical guidelines to triage for the present study (which were based upon actual Dutch guidelines). Here, the expert was also motivated to ask clarifying questions at any time.

Then, the expert was asked to conduct triage in the implemented testbed without the help of an artificial agent. In this baseline condition, 16 patients had to be triaged. The expert was instructed to apply the given ethical and medical guidelines. After completion of the baseline task, questions addressing the ecological validity were administered (as proposed in Section 6.2.3).

The expert was then asked to triage a new set of 16 patients, this time receiving decision advice from their personal artificial agent according to TDP-1. During the task, instructions were given to think aloud (TA), which was recorded while notes were taken. When the expert considered the guidelines to be indecisive or inappropriate, instruction was given to follow their own personal moral values and decide accordingly. After completion of the task, subjective measurements were taken concerning *experienced control* and the *value* and *usefulness of explanations* to serve as input for the semi-structured interview afterward. This process was repeated for TDP-2 and TDP-3.

When all conditions were completed, the expert was asked to reflect on all three TDPs. An indication had to be given which TDP they would prefer to use in their work (if at all), and why.

After the interviews, all collected data was anonymized using pseudonymization and a key-file that was removed after 2 weeks.

### 6.3 Results

The findings per TDP will be reported as follows:

• *Team collaboration:* The participating expert and the agent jointly performed the assigned task of assigning medical care to a set of patients. The nature of this human-agent collaboration was shaped by the particular TDP. Per TDP we report on how the expert evaluated the collaboration and the task division.
• *Control:* Per TDP we report if the domain experts experienced to be in sufficient control to ensure that all decisions were made according to their own personal moral values. For this, we used results from questionnaires and interviews, seeking for trends in how much control the expert experienced.
• *Explanations:* For humans to exercise control efficiently and accurate, they need to have an understanding about their agent partners which explanations can help ascertain. Outcomes from the explanation questionnaires and semi-structured interviews were used to report how the experts evaluated the agent’s explanations, and if they supported a better understanding of the team.

#### 6.3.1 Ecological Validity

The domain experts scored the ecological validity of the used scenario in which medical triage was conducted and the available information for doing so with an average of 75.71 (*SD =* 10.50) out of 100. They scored the ecological validity of the stressors in the task with 76.43 (*SD =* 6.39) out of 100. Two respondents indicated that the time pressure induced by the pace of patients being submitted for triage was too high. We take these findings as a reassurance that the developed testbed and task is suitable for investigating MHC in a situated manner.

#### 6.3.2 Findings TDP-1

In this design of human-agent team collaboration, the agent provides information and gives advice with the human making the actual triage decision.

##### 6.3.2.1 Team collaboration

The domain experts evaluated this pattern of collaboration with the agent fairly positively. Three out of the seven experts preferred TDP-1 over the other two Team Design Patterns. Two out of the seven experts mentioned TDP-1 in combination with TDP-2 as their ideal collaboration with an intelligent system. Furthermore, they pointed out that the agent did what computers are best at, discovering and presenting statistical relationships in the domain; and that they themselves could concentrate on making decisions. One interviewee said: “The agent provided quick computational power to calculate valuable data, whereas I as a human could make the actual moral decision.”

The interviewer asked each expert how they experienced the role of the decision support agent. They indicated that if the agent’s advice corresponded to their initial opinion, the congruence was regarded as a confirmation that it was an appropriate decision. If, however, the agent’s advice deviated from the own opinion, then this was for many the sign to change their decision. Overall the experts elaborated that they interpreted the agent’s advice to be representative for what doctors in general decide. One expert argued: “all those other doctors probably know best.”

##### 6.3.2.2 Control

The domain experts found the task with the decision support difficult and strenuous. Most experts pointed out during the interview to feel responsible to assign the best possible care to all patients, which aligned with the results of question 3 of Table 5. Four experts scored a “totally agree” on the experienced responsibility over the patients well-being. The other three experts scored this with a “very much agree.” They said to realize that a swift processing was important, as to prevent deterioration of a patient’s condition pending their triage decision, subsequently experiencing stress about this. On being asked whether they judged this as a threat to maintaining control, most experts indicated to be able to cope with the time pressure. They said that the support offered by the agent (such as computational information about a patient’s survival chances for every possible care) helped them to manage the imposed time pressure. Overall, the experts reported to experience adequate control over the process and decision making. Furthermore, all evaluated their triage decisions to be compliant with their own moral values and the provided ethical guidelines.

##### 6.3.2.3 Explanations

The predominant response of domain experts was that the conditions imposed by the experimental simulation did not allow them to form a proper judgment about the value of the explanations. They felt to be working under extreme time pressure (*see* above), which precluded them to process the explanations. One expert remarked: “all that text took too much time to read,” and suggested to provide explanations in a visual form instead. Another expert indicated that the assumption that “the agent acted as a representative of other human doctors,” allowed him to disregard the explanation altogether. Ironically, one purpose of explanations in human-agent teams is to establish and support *appropriate trust* in each other. Thus, during the interview the experts mentioned to be unable to conduct a proper evaluation of the given explanations. However, in response to the questions about the value of explanations, they rated the explanations as neutral to positive for achieving a better understanding. Here, an average of 3.38 (*SD = 0.49*) was given to the usefulness of explanation types where 1 was considered as “Strongly disagree” and 5 as “Strongly agree” (see Section 6.2.3).

#### 6.3.3 Findings TDP-2

In this design of human-agent team collaboration, the agent proposes which patients to assign to the human and which patients to the agent. This is done based on an earlier value elicitation process using a questionnaire. The agent can provide an explanation for its intended decision as well as the allocation of each single patient. The human can overrule this allocation. Each patient is independently triaged by both human and agent based on this (overruled) allocation.

##### 6.3.3.1 Team Collaboration

The interviewer asked the domain experts how they experienced the role of the dynamic task allocation agent. They indicated that the division within the triage task helped them focus on their own patients. Also, they experienced the task to go *faster* in comparison to TDP-1, which was evaluated as a pleasant effect. Two experts mentioned that reviewing the explanation took too long and would have a detrimental effect on task performance. Instead, they kept patients allocated to the agents without reviewing the relevant explanations.

All experts mentioned to *trust* the agent in the decisions it made for the patients that were assigned to it. The overall motivation was that they understood and accepted why and how the agent assigned patients. To quote one expert; “I understood why the intelligent system assigned certain patients to itself (…), and that its decisions were based on my value elicitation.”

##### 6.3.3.2 Control

During the interview most domain experts indicated that they considered it a challenge to maintain an overview of the patients requiring a triage decision. One expert rated complete agreement (5/5), and four a strong agreement (4/5) on question 1 of Table 5 of not being able to keep an overview. These expert argued that they felt a need to continuously monitor all patients, including those assigned to the agent. This required too much effort according to them, to exercise adequate control. When asked to elaborate on this, they argued that the agent’s triage decisions (e.g., assigning a patient to the IC) had an impact on their own decision space (e.g., all available IC-beds occupied). Keeping overview on what the agent was doing, while simultaneously paying attention to their own patients often imposed too much pressure to exercise adequate control, as three experts emphasized explicitly.

Opposed to the five experts experiencing high workload, the other two experts reported to not feel this pressure. They explicitly reported to rely on the qualities of the agent and its triage decisions. When asked what caused this reliance, they argued that they had noticed the agent to comply to the their personal moral values, assessed earlier during elicitation. This resulted in a feeling for them that decisions could be safely dealt with by the agent. Which in turn made the experts experience more time available to focus on assigning care to their own patients.

On average the experts felt slightly less responsible for the patient’s well-being compare to TDP-1 (TDP-1 scored an average of 4.58 ((*SD = 0.49*) and TDP-2 scored an average of 4.16 (*SD = 0.68*) on question 3 from Table 5).

All experts were positive about the option to overrule the agent’s assignment. This was evaluated as a valuable asset of this TDP and they all indicated that it contributed to their experienced control.

##### 6.3.3.3 Explanations

Five of the experts mentioned that they *missed* the statistical data that was presented in TDP-1. They interpreted this data as an explanation of agent reasoning, even though it was not presented as such.

Similar to the previous condition, the view on how the explanations were presented was referenced by all experts. Again, it was suggested that visualizations might be beneficial, since the provided text took them too much time and effort to interpret.

Overall, the explanations were not utilized excessively, as four experts reported. However when they felt they had the time, they were perceived as helpful, establishing a form of *trust* and *understanding* of the system. The open-ended interview question on whether the experts considered the explanations as important, all answered with “yes.” One expert indicated that: “The explanations help me during the task. If these would not be provided, it would have been very unpleasant.”

#### 6.3.4 Findings TDP-3

In this design of human-agent team collaboration, the agent autonomously makes all decisions swiftly based on the elicited moral values elicited before the task. The human observes the agent making these decisions to understand how the elicited values impact agent behavior and to make adjustments next time if needed. More information about the agent’s reasoning behind a decision could be requested. Note that this collaboration does not allow the human to exercise instantaneous control such as intervening in an agent’s decision.

##### 6.3.4.1 Team Collaboration

All experts reported this collaboration as uncomfortable during the interview. Two explicitly motivated this by the fast pace patients entered the environment, and two with not being able to overrule the agent. In some cases, experts were not motivated to request an explanation on why certain decisions were made. One argued: “I do not feel part of a team, because I don’t play a role in the decision making process.” As a result, the experts did not feel responsible for the decisions made by the agent, similar as in TDP-2.

##### 6.3.4.2 Control

In all cases, the experts stressed the discomfort that arose from not having the opportunity to overrule the intelligent agent. When asked about their *trust* in the agent, two experts responded that the agent was compliant to the earlier given value elicitation. Three also mentioned they understood the reasoning of the agent, which also established trust. Interestingly, one mentioned that this did not meant that (s)he always agreed with the triage decisions made by the agent.

Experts did not feel motivated to take on their supervisory role in the collaboration. This was reported by the same four experts who noted a high pace and time pressure. When asked, their reason was the stress and lack of overview evoked by this pace.

##### 6.3.4.3 Explanations

The two experts who reported on the uncomfortable high pace, indicated to seldomly read the explanations. One of them commented: “If I read one explanation, I miss out on three other patients and the decision made for those.” The five experts who indicated they did read the explanations, scored on average 3.67 (*SD = 0.75*) to question 5 of Table 6. Indicating they found the explanations useful in understanding the agent’s reasoning.

The interviewer asked these five experts about the trigger for wanting more information about the agent’s reasoning. Two explained that this was only when they did not agree with the decision made by the agent. They expressed a curiosity in *why* the agent would make such a decision to be able to better understand the system. The other three explained to have an overall curiosity, independent of the made decision.

#### 6.3.5 Comparative Findings

This section highlights similarities and differences within the three collaboration designs (summarized in Table 8):

**TABLE 8:** A summary of the key findings separated on the three aspects measured and the Team Design Patterns tested. In TDP-1 the agent provides advice to an expert making decisions. In TDP-2 the agent distributes decisions between itself and the expert, which can be overwritten. In TDP-3 the agent made all decisions under supervision according to elicited decision rules beforehand.

Aspect	General findings	TDP-1	TDP-2	TDP-3
Team collaboration	Valued the more direct control was experienced	Most valued due the direct control	Mostly valued for its high potential	Did not feel like a collaboration
Sense of control	Only when capable of influencing decisions directly	High degree due to agent not making any decisions	Some experienced control, due to feeling capable of intervention	No feeling of control, as it was a delayed form of control
Use of explanations	Perceived as useful in hindsight, but not actually used	Intermediate statistics were found most valuable	Explanations were not used or ignored	Observing behavior more useful than explanations due to agent decision speed

##### 6.3.5.1 Team Collaboration

Overall, all interviewed domain experts reported TDP-1 as their preferred collaboration design. They substantiated this preference by the clear division between human and machine, which was appreciated.

Notably is that four out of seven experts motivated their interest in TDP-2, especially when the agent would be “more mature,” as one expert described it. When asked to elaborate on this, they referred to the agent in TDP-1 who provided additional information as well as advice. Effectively, they proposed a combination of the data-driven decision support agent from TDP-1 with the dynamic task allocation functionality of the agent in TDP-2, implying that the data-driven agent would make its own decisions.

In all three conditions, the speed of incoming patients was emphasized. Interestingly, in TDP-1 and TDP-3 this was perceived as unpleasant, while in TDP-2 the speed of assigning patients as a team was being appreciated. In fact, in this condition one expert mentioned to deliberately leave patients assigned to the agent, as to make sure those patients received care earlier.

Furthermore, there was a sense of confirmation among all experts in TDP-1 and TDP-2. They experienced it as helpful when the advice (TDP-1) or decision (TDP-2) was congruent with their own initial decisions. Within TDP-1, they felt supported by the agent rather than collaborating with it. As such, TDP-1 did not result in a feeling of collaborating with the agent. The supervised autonomy collaboration from TDP-3 received the least willingness from experts to collaborate, since they felt they could not take part in the decision making process.

##### 6.3.5.2 Control

A comparison of the results per TDP revealed that TDP-1 was favoured by three experts when it came to the *sense of control*. Furthermore, when the interviewer asked to rank their top three of the TDPs, TDP-1 was placed first by five experts. The main reason for this was their sense of having complete control over decision making. In contrast to TDP-1, TDP-3 was the least preferred by six experts due to the inability to directly influence the decision making process. The seventh expert who favored this TDP, only did so under the condition that the human team member would receive the ability to overrule the agent.

Even though *speed* was mentioned to be an asset in TDP-2, it also effectuated a lack of overview. Because both expert and agent could influence the environment, all experts had difficulties keeping track of the situation. However, they did experienced control as they could influence on the task allocation. In other TDPs, increased collaboration speed resulted in increased time pressure, overall resulting in experiencing less control.

Furthermore, none of the experts experienced the ability to exercise control through the value elicitation process, which determined the agent’s decision making in TDP-2 and TDP-3. At times, they mentioned that they felt the agent did comply to the elicited moral values, but this did not result in an experience of being in control. This was especially the case in TDP-3, where intervention was not possible as was the case in TDP-2.

Lastly, TDP-3 evoked the least feeling of responsibility over patients in comparison with TDP-1 and TDP-2. The lack of experiencing control through value elicitation over the agent negatively impacted the sense of responsibility for the agent’s decisions.

##### 6.3.5.3. Explanations

In general, explanations were found useful, as all experts mentioned in the semi-structured interview, but mostly in retrospect as they were utilized only occasionally in TDPs. The main reason for not requesting an explanation was the experienced time pressure.

The most common trigger for requesting an explanation from the agent was when the agent showed incongruency with the expert’s initial own decision. In those cases, it established a form of *trust* as well as better *comprehension* of the system according to the experts.

During TDP-3, all experts felt overwhelmed by the agent’s decision speed and felt they learned more from observing the agent than by reading explanations.

## 7 Discussion

The advancements of embedded artificial intelligence allows modern technology to conduct complex tasks more and more. This provides new opportunities, but it also raises the question whether and how humans can still exert meaningful control over the technology’s behavior. This is especially important for tasks for which ethical and moral values apply. To address this question it is important to first define multiple manners of organizing the contribution of humans and technology in human-agent teams. Subsequently, research is needed into how these different patterns of human-agent collaboration affect the human’s control over the agent’s behavior and the team’s performance. This study addresses both needs.

Three different design patterns for human-agent teaming have been developed and implemented into a medical triage task. Then, medical domain experts were asked to work with these agents under these team design patterns. Multiple qualitative methods and measures were used to learn how the domain experts experienced the collaboration with the agents, and, in particular, how well they felt in control over the task and over the decisions taken by the team. We feel that this kind of qualitative research is important to obtain a better understanding of how different teaming options affect the feasibility of humans to exert meaningful human control. This understanding is needed to define and refine the team design patterns for use in future practical applications.

Findings indicated that the domain experts wished to make as many decisions as possible even when experiencing an already high workload. Furthermore, they only felt responsible for their own decisions instead of all decisions made within the team. As such we identify several challenges in the design of a human-agent team based on these finding. First, the way humans and agents collaborate should ensure that humans feel responsible for agent behavior, otherwise they will lack the motivation to exercise the necessary control. Secondly, humans need to be prevented from exercising control unnecessary often when they do feel responsible as this will increase their perceived workload. This includes protecting humans from their tendency to take on too many decisions. These two challenges could be solved by making agents more aware of the human’s mental state to adjust the way they collaborate (e.g. through physiological measurements or lack of response time thresholds).

Several findings indicated that the experience of control depends on how immediate its observed effects were. Two control mechanisms were evaluated varying in how instantaneous their effects were, with the domain experts favoring the more instantaneous control (task reallocation) over the other (iterative value elicitation). Depending on the task, instantaneous control is not possible or not doable for humans. When a effects are delayed, agents could explain the consequences of the exercised control to humans. This “consequential explanation type” could improve the experienced control as well as support the human assessment if the exercised control would have the intended effects. These type of explanations could for instance include simulations of agent behavior given the intended control signal.

Different types of explanations were used in the various collaboration designs. Their purpose was to improve control and calibrate trust by enabling an accurate mental model of the agent’s reasoning. However, findings indicated that the experts almost never reviewed the explanations due to the experienced time pressure caused by their desire to decide quickly. Interestingly they did value all the explanations in retrospect and could see their potential. This shows that research into agent-generated explanations should not only focus on their content but put equal, if not more, focus on how and when they are presented. Ideally, the agent should be made aware of human workload and adjust its explanations accordingly. This again underlines the need for agents to be aware of the human’s mental state, not only to adjust how they collaborate but also how they explain.

Some findings indicated that the domain experts trusted the agents too much, and relied at times more on the agent’s judgment then their own. Even though they were instructed to be critical as they were held responsible for the agent’s behavior. This indicates a potential automation bias. This would explain why they did not feel the need to make time to review and interpret the explanations. Interestingly, the explanations were intended, among others, to counter this over trust. Within the field of Explainable AI it is often mentioned that explanations support appropriate trust calibration. However, if too much trust prevents humans from interpreting the explanations, those explanations become meaningless. Further research on the relation between automation bias and the use of explanations is warranted. If this hypothesis proves to be true, agents need to be aware how much their human partners rely on them and adjust their way of presenting explanations accordingly.

A limitation of the study is its qualitative nature to evaluate the different collaboration designs. Although, as we argued, a qualitative study is better suited in exploratory research as it provides more information compared to a quantitative study. However, only one task was used with specific properties, making it difficult to generalize the results. Future studies should focus on further similar evaluations with tasks combining both objective and subjective measurements. Research could expand our designs to include agents who model and track the human mental state and adjusts their collaboration and explanations accordingly. Specifically, further study is warranted on how agents can foster a human feeling of responsibility and facilitate the experience of control when its effects are delayed. Finally, it is important to research how agents should formulate and present their explanations such that humans feel they have the time and need to review them.

## 8 Conclusion

This paper addresses the design of the collaboration in a human-agent team, specifically of that in morally salient domains where humans should have meaningful control over the agents. This control needs to ensure that team behavior is compliant to human moral values and ethical guidelines. Otherwise, the human should be able to be held responsible. Three of such teams designs were presented. Each varied in the agent’s autonomy and we evaluated the experienced control and the value of provided explanations in each using structured-interviews with domain experts.

These three design patterns and the performed interviews form a first iteration toward designing human-agent teams that support meaningful human control for various tasks. A design pattern approach was taken, and a first set of measurements were introduced together with a reusable testbed to evaluate human-agent collaboration to support meaningful human control.

Results from the expert interviews showed that the used task of medical triage was sufficiently realistic and its simulation valid. Furthermore, we found that how responsible humans feel for agent decisions relates to their involvement in those decisions. If the experts only supervised they did not feel responsible, even though they could exercise control by defining agent behavior beforehand. The more the experts felt in collaboration with agents, the more they felt in control. With having sufficient time and influence over the agent as prerequisites for this collaboration. To support this, agents could benefit from monitoring the workload of humans and adjust their collaboration form and explanations accordingly. Specifically when humans experience time pressure, agents could motivate humans to remain involved in their decisions as they can be held responsible for them. In addition, agents need to adjust when and how they communicate their explanations in these cases to also motivate humans to review their explanations. As the results from our interviews showed that pressured humans might tend to trust the agent too much and ignore its explanations designed to prevent such over-trust. This study was a first step in exploring how domain experts experience human-agent team designs that aim to enable meaningful human control. The identified trends in expert opinions provide valuable future research topics within the field of human-agent teaming.

## Data Availability

The original contributions presented in the study are included in the article/Supplementary Material, further inquiries can be directed to the corresponding author.

## Footnotes

1 The task’s implementation is available on request by contacting the corresponding author.

2 The implementation of both the task and agents is available under the MIT License (2020) and can be found on this link: https://github.com/matrx-software/MHC-for-triage-agents.
